# Phytochemical analyses, antimicrobial, antioxidant and anticancer activities of endophytic fungi from leaves of *Reseda pruinosa*

**DOI:** 10.1371/journal.pone.0357606

**Published:** 2026-10-29

**Authors:** Amr H. Hashem, Nosiba S. Basher, Abeer S. Aloufi, Salem S. Salem, Mahmoud M. Fawzi, Mohamed M. Abdelaziz, Ahmed S. Abdelrahman, Mohamed S. Khader, Abdallah E. Emam, Mohamed A. Goma, Ahmed R. Salama, Nasir A. Ibrahim, Sulaiman A. Alsalamah, Fahd A. Nasr, Amer M. Abdelaziz

**Affiliations:** 1 Botany and Microbiology Department, Faculty of Science, Al-Azhar University, Cairo, Egypt; 2 Biology Department, College of Science, Imam Mohammad Ibn Saud Islamic University (IMSIU), Riyadh, Saudi Arabia; 3 Department of Biology, College of Science, Princess Nourah bint Abdulrahman University, Riyadh, Saudi Arabia; 4 Faculty of Science, Al-Azhar University, Cairo, Egypt; University of Bari Aldo Moro: Universita degli Studi di Bari Aldo Moro, ITALY

## Abstract

Fungal endophytes considered one of the major sources of promising bioactive compounds. This study aimed to isolate endophytic fungi from leaves of *Reseda pruinosa* for the first time, and to evaluate their biological activities. Results illustrated that, two fungal endophytes isolates were isolated, identified as *Alternaria* sp. and *Alternaria alternata* morphologically and genetically. Phytochemical analyses of these two fungal isolates metabolites showed significant amounts of phenolics, flavonoids, tannins, and alkaloids. GC-MS analyses of the crude extracts of these two fungal isolates showed that, major compounds in these extracts belong to the classes of fatty acid esters, fatty acids, and terpenoids. Results revealed that the MCF-7 cancer cells was susceptible to the anticancer effects of both fungal isolates crude extracts, with IC_50_ values of 96.3 and 117.4 µg/ml, respectively. Additionally, it showed promising antibacterial efficacy against pathogenic bacteria and fungi. Moreover, both crude extracts of fungal isolates exhibited antioxidant activity, with EC_50_ values of 234.8 µg/ml and 133.4 µg/ml respectively. Endophytic *Alternaria* species from leaves of *R. pruinosa* are a novel source for bioactive compounds that used in different medical fields.

## 1. Introduction

Endophytes are microorganisms that live in tissues under the epidermal cell layers and have no discernible negative impact on the host [1]. Fungi are the most often isolated endophytes. Fungal endophytes establish a mutualistic relationship with host plants, often providing benefits such as enhanced nutrient absorption and increased plant resistance to pathogens [2]. Endophytes live in intercellular gaps or within plant cells, and are most typically found in roots, stems, leaves, and seeds [3]. Endophytes can confer various benefits to the host plant, such as increased absorption of nutrients, stress tolerance, and defense versus pathogens and herbivores [4–8]. Fungal endophytes play important ecological roles, where help plants adapt to environmental stresses, such as drought, high temperatures, or nutrient deficiencies [9]. Furthermore, fungal endophytes can influence plant community dynamics and nutrient cycling in ecosystems. Numerous secondary metabolites, including as peptides, phenolics, terpenoids, and alkaloids, are produced by fungal endophytes [10]. These bioactive substances are capable of a wide range of biological functions, such as antimicrobial, antifungal, insecticidal, and anticancer properties [11,12]. Some of these metabolites have been investigated for their potential pharmaceutical and agricultural applications [4,13]. *Alternaria* is the fungal genus described as ubiquitous fungi and they are up to 300 species, which are classified into 26 species according to the genetic and morphological characteristics [14]. Endophytic *Alternaria* is considered a promising agent that produces bioactive metabolites that are more bio-effective than the secondary metabolites that are produced by saprophytic *Alternaria* [15]. *Alternaria* is a fungal endophyte that contains a reservoir of bioactive metabolites that possess medicinal, pharmaceutical, and agricultural applications [16,17]. The bioactive compounds synthesized by *Alternaria* fungi fall into a number of groups: steroids, alkaloids, flavonoids, tannins, phenolic acids, and terpenoids [18]. *Reseda* has been described to have antitumor, anti–HIV, cytotoxic, antimicrobial, and anti-inflammatory activities [19]. *Reseda pruinosa* was selected in the current study for isolation of fungal endophytes which live inside its tissues, where no previous reports about this point. *Reseda pruinosa* was selected due to it has been used in traditional medicine and pharmacology for its potential therapeutic properties as an anti-inflammatory, antiseptic, analgesic, and has unique habitat [20]. This study aimed to isolate fungal endophytes from *R. pruinosa* leaves for the first time. Additionally, to evaluate their phytochemicals, antimicrobial, antioxidant, and anticancer activities.

## 2. Materials and methods

### 2.1. Source of plant sample

Healthy *R. pruinosa* plant was collected in disinfected polyethylene bags in an ice box (4°C) from the plant garden at the Faculty of Science, Al-Azhar University, Cairo, Egypt, and identified with the assistance of Prof. Abdou M. Hamed, Professor of Plant Ecology, Botany and Microbiology Dept., Faculty of Science, Al-Azhar University.

### 2.2. Isolation of fungal endophytes

The fungal endophytes were isolated following the method described by Attia et al. [21]. Washing healthy *R. pruinosa* leaves with tap water, sterilizing them with ethanol (70%) 30 seconds, then immersed in NaOCl (4%) for 2 minutes, then 70% ethanol for 30 seconds. The plant parts that had been sterilized were put on PDA medium (Oxoid) along with 0.2 g/L of the antibiotic chloramphenicol. The PDA plates were incubated at 27˚C, for 21 days, with observed every day with a stereomicroscope.

### 2.3. Identification of endophytic fungi

Fungal isolates, were morphologically identified based on characteristics such as color, texture, colony diameter, mycelium, and conidia. The isolated fungal endophytes were identified utilizing molecular identification [22,23]. The genomic DNA was extracted and purified using the Quick-DNA Fungal Microprep Kit (Zymo Research; D6007), with molecular identification performed via the internal transcribed spacer (ITS) region. The primer sequences used for this process were ITS1-F (5’-TCCGTAGGTGAACCTGCGG-3’) as the forward primer and ITS2-R (5’-TCCTCCGCTTATTGATATGC-3’) as the reverse primer. PCR products were purified using the GeneJET PCR Purification Kit and sequenced with an Applied Biosystems kit. The extraction process involved mixing 200 µL of fungal liquid media with water, solid tissue buffer, and proteinase K, followed by incubation at 55ºC for 2 hours. The mixture was centrifuged, and the supernatant was transferred to a new tube. After adding genomic binding buffer, the mixture was processed through a Zymo-Spin™ column, with multiple washing steps using specific buffers. Finally, elution buffer was added, incubated, and centrifuged to obtain purified DNA.

The resulting sequences analyzed using the BLAST algorithm from the National Center for Biotechnology Information (NCBI) database to identify closely related phylogenetic sequences. The fungal isolates, were subsequently deposited in the GenBank database with unique accession numbers. For constructing the phylogenetic tree, the neighbor-joining method from the MEGA 5.0 program employed using 500 bootstraps.

### 2.4. Extraction of bioactive compounds from fungal metabolites

Fungal endophytes were cultivated on PDB (Oxoid) at a temperature of 27 ± 2˚C for 21 days [24]. The fungal metabolites were filtered and extracted using ethyl acetate. After homogenizing the fungal filtrate for ten minutes with an equal amount of ethyl acetate solvent and vortexing it using a vortex shaker, the mixture was then developed for an additional 5 minutes until two distinct clear layers were created. A rotary evaporator (Heidolph VV2001, Germany) was utilized to evaporate the constructed ethyl acetate layer at 45 ^o^C. After that, DMSO, was used to dissolve the fungal ethyl acetate extract, and preserved at a temperature of –20 ^o^C for further analysis.

### 2.5. Quantitative screening of fungal phytochemicals

The total phenolic analysis was conducted utilizing the methods of [25]. And total flavonoids were carried out according to the method used by [26]. The total tannin content was determined using the method proposed by [27]. Assayed total alkaloid using the [28] technique.

### 2.6. GC-MS analysis

The chemical composition of fungal extracts were identified by gas chromatography–mass spectrometry (GC–MS) using a Thermo Scientific Trace GC 1310 gas chromatograph equipped with an ISQ mass spectrometer (Thermo Scientific, Austin, TX, USA). Chromatographic separation was performed on a TG-5MS fused-silica capillary column (30 m × 0.25 mm i.d. × 0.25 μm film thickness). The oven temperature was initially set at 40°C and then increased at a rate of 5°C min-1–120°C, where it was held for a short period of time before increasing to 280°C. The final temperature was held for 1 min to ensure complete elution of the analytes. The analysis was carried out with helium as carrier gas at a constant flow rate of 1.0 mL min ^−1^.The automatic sample injection was performed using the AS1300 autosampler. Injection of 1 μL of each diluted extract was performed in split mode with a solvent delay of 3 min. Temperatures of injector and transfer line were 250°C and 260°C, respectively. Mass spectral data were obtained in full scan mode over a mass-to-charge (m/z) range of 40–1000 in electron ionization (EI) at an ionization energy of 70 eV. The identification of the detected constituents was performed by comparing the mass spectra and retention characteristics obtained with those available in the WILEY 09 and NIST 11 mass spectral libraries, which allowed for the tentative identification of the chemical compounds present in the fungal extract [29].

### 2.7. In-vitro cytotoxicity

MTT stain was used for determination the cytotoxicity of extracts of *Alternaria* sp*.* and *A. alternata* at concentrations of 1000 - 7.81 µg/mL [30]. Normal Vero cell line was used for evaluation the safety of these crude extracts, and cancerous MCF7 cell line was used for assessment the anticancer activity of these crude extracts at safe concentrations. The cell viability of Vero and inhibition percentages of MCF7 cells were calculated as indicated in Equations 1 and 2 as follows:


$$\begin{array}{c} \text{Viability }\%=\;\frac{\text{Test OD}}{\text{Control OD}}\text{ X 100} \end{array}$$ (1)

$$\begin{array}{c} \text{Inhibition }\%=\text{100}-\text{Viability }\% \end{array}$$ (2)

### 2.8. Antimicrobial activity

The antimicrobial efficacy of crude extracts from *Alternaria* sp*.* and *A. alternata* was tested versus a variety of pathogenic fungi and bacteria. The pathogenic microbes include *E. coli* ATCC 25922*, P. aeruginosa* ATCC 27853*, S. aureus* ATCC 25923*, B. subtilis* ATCC 6051*, C. albicans* ATCC 90028 and *C. neoformans* ATCC 14116. These were cultured on MHA and PDA media based on the microbe type. Crude extracts, amoxicillin/clavulanic acid, and fluconazole were each placed in separate 8mm wells (100 µl) and incubated at 4 °C for 24 h. After that, each plate was incubated for 24 h at 37 °C for bacteria and 48 h at 28 °C for unicellular fungi. Following incubation, the inhibitory zones were detected and noted [31–33].

### 2.9. Antioxidant activity

The antioxidant activity of crude extracts from *Alternaria* sp. and *A. alternata* was carried out utilizing DPPH method [18]. Crude extracts were evaluated for their ability to scavenge DPPH radicals at doses ranging from 1000 to 7.81 µg/mL. At 517 nm, each concentration’s absorbance was compared to the standard (Ascorbic acid). The standard utilized was ascorbic acid. The following formula was used to determine the antioxidant activity:


$$\begin{array}{c} \text{ DPPH scavenging activity }(\%)=\frac{\text{Control Abs }-\text{ Extract Abs}}{\text{Control Abs}}x\;\text{1}\text{0}\text{0} \end{array}$$ (3)

## 3. Results and discussion

### 3.1. Endophytic fungal isolation and identification

Two fungal isolates were isolated from the sterilized *R. pruinosa* leaves. Morphological identification revealed that two fungal isolates belonged to the genus *Alternaria* (Deuteromycota), which is consistent with Crozier, Thomas [34] where they noted Deuteromycota’s widely distributed status as endophytic fungi. Isolate R1: Colonies on a PDA grow up to 50 mm in dimension in 7 days at 28°C with deep black mycelium and reverse black. Conidia are short and irregularly circular, septating both laterally and longitudinally with up to two transverses and two longitudinals. Conidia are usually of circular in overall shape. Isolate R2: Colonies on a PDA with white to gray-brown mycelium can grow to 50 mm in dimension in 7 days at 28°C. Reverse the colors brown and dark in the center. The conidia are small and irregularly shaped, with septa occurring both laterally and longitudinally, containing up to six transverse and two to three longitudinal or oblique septa. Conidia often have a club-shaped appearance, narrowing towards the tips and developing a little beak. To validate the identification based on morphology, two fungal strains were identified genetically, as shown in Fig 1. Molecular identification revealed that two fungal strains, R1 and R2, were identified as *Alternaria* sp. and *A. alternata* with similarity percentages of 99.29% and 99.64%, respectively, and both were deposited in the Gene-Bank with accession codes OQ064057 and OQ064058. Recently, endophytic *Alternaria* species were isolated from different parts of plants: *A. alternata* was isolated from *Picrorhiza kurroa* [35], leaves of *Ziziphus spina-christi* Elghaffar, Amin [36], *Vitex rotundifolia* [37] *Azadirachta indica* (*A. Juss*) [38] and leaves of *Calotropis procera* [39].

**Fig 1:**
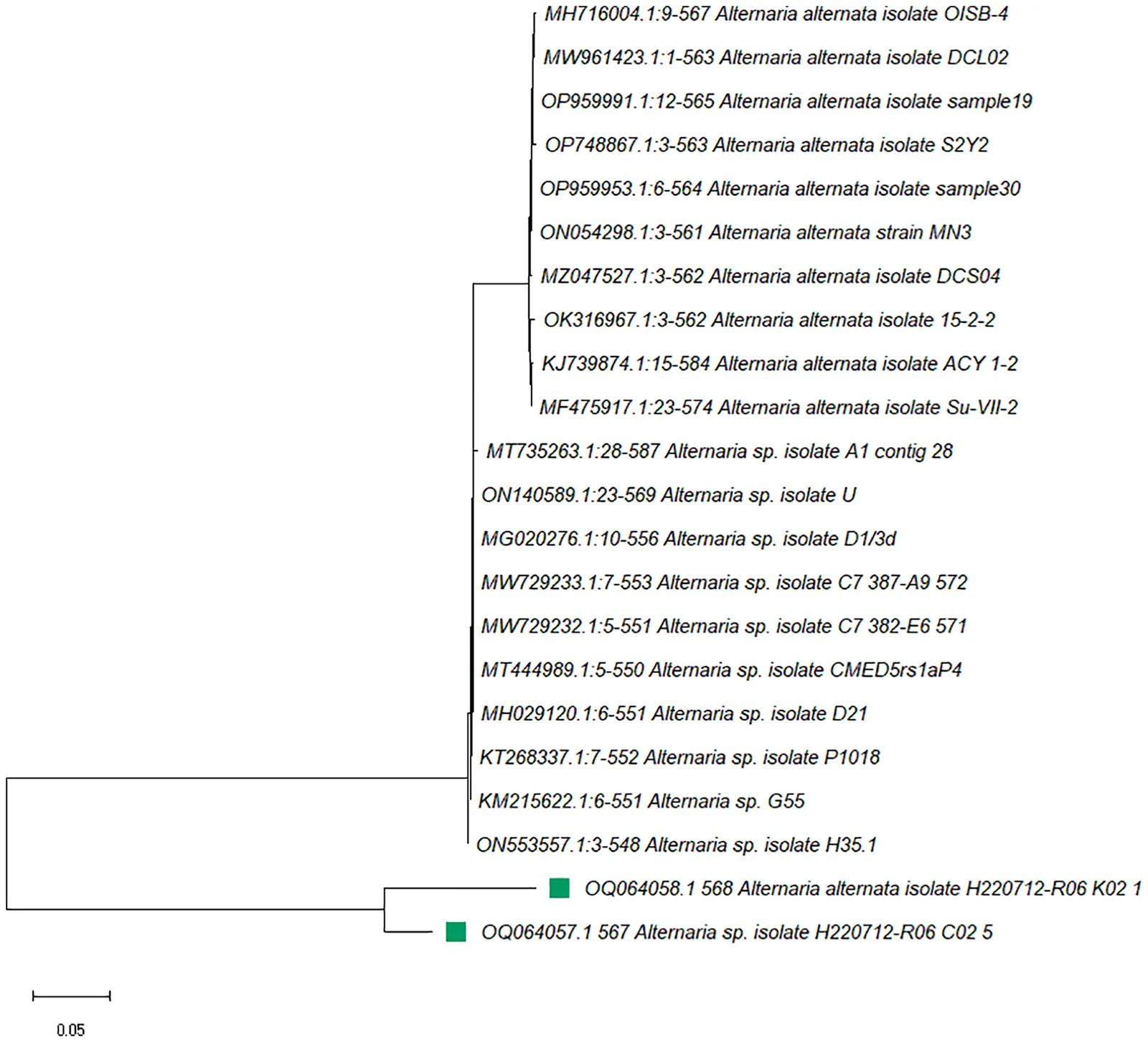
Phylogenetic tree of molecularly identified *Alternaria* sp. and *A. alternata with* accession codes OQ064057 and OQ064058 respectively.

### 3.2. Phytochemical analysis

The occurrence of fungal metabolites highlights the significance of natural chemicals as advantageous agents. Both fungal isolates can generate significant quantities of phenolics, flavonoids, tannins, and alkaloids as shown in Fig 2. The findings are consistent with multiple studies indicating that ethyl acetate-extracted endophytic fungi contain a variety of physiologically active compounds, including flavonoids, phenolics, tannins, and alkaloids, which hold potential applications in both human health and agricultural sectors [40–42]. The fungal endophytes *Alternaria* sp*.* and *A. alternata* had phenolic levels of 694 and 267.7 mg/g, respectively. The results showed a direct positive relationship between the phenolics in the endophytic fungal ethyl extracts and the bactericidal activity. Fungal endophytes’ bactericidal activity against human pathogens was explained by their high phenolic content [43]. Phenolics had highly selective antibacterial effects on human pathogens while having no negative effects on probiotics [44]. More specifically, the antioxidant activity of phenolics, along with other mechanisms, may contribute to human health as a novel alternative to food safety [45]. Phenolic compounds are reactive metabolites that, through the elimination of free radicals and the chelation of metal ions, can catalyze lipid oxidation [46]. *Alternaria* sp*.* and *A. alternata* had the same flavonoid levels of 26.9 µg/mL. Flavonoids are documented to have a wide variety of biologically active properties that can have helpful properties for human general health, including inflammatory depression, anti-cancer, antioxidants, enzyme inhibitors, hormone regulators, immune modulators, therapeutic values, and antiviral properties [47]. The high antioxidant achievement of flavonoids is explained by several mechanisms, including destruction of ROS development through inhibition of enzymes, removal of free radicals, and guidelines for antioxidant human defense [48]. Their mode of bactericidal inaction may be due to their capability to deactivate microbial connections, disrupt bacterial membranes, and have cell transference properties. They also help in the reserve of several enzymes associated with bacterial and viral virulence. Flavonoids can prevent cancer diseases by affecting the beginning and advancement stages of the cancers, along with their impacts on progress and hormonal action [49]. Tannins had a positive correlation with human probiotics and human health benefits; thus, they were applied as food enrichment [50]. The results demonstrated that the tannins produced by *Alternaria* sp*.* and *A. alternata* recorded 12.3 and 33.9 µg/ml, respectively. Tannins are considered a hopeful bactericide for avoiding bacterial pathogen infections by iron chelation, bacterial wall creation inhibition, disorder of the bacterial membrane, and a reserve of bio–synthetic fatty acids [51]. Treatments involving tannin metabolism are gaining popularity for utilizing the impact of tannins on biology in drug development and advantages for health. Tannins possess valuable pharmacological properties, including anti–diabetic, anti–oxidant, anti–inflammatory, anti-cancer, and cardioprotective effects [52]. The bactericidal properties of tannins can help prolong the shelf life of specific foods. Tannins have demonstrated several physiological effects, including accelerating blood coagulation, reducing blood pressure, decreasing serum lipids, and regulating immunological responses [53]. Moreover, results illustrated that the alkaloids produced by *Alternaria* sp*.* and *A. alternata* ethyl acetate extract have 12.3 and 33.9 µg/ml, respectively. Natural alkaloids have the potential to be used as bactericidal agents against *S. aureus*, including MRSA, *Streptococcus pyogenes, Shigella strains, Campylobacter* sp*.,* and *S. aureus*, as well as antiviral, fungicidal, and anticancer agents in human cervical cancer Hela cells [54]. Endophytes are a reservoir for numerous secondary metabolites such as bactericidal, and fungicidal compounds for agricultural and human use [21,41,55,56]. *Alternaria* endophytes have the capability to create hopeful secondary metabolites as well as alkaloids, terpenoids, sterols, vitamins, and terpenes with different bioactivities [18,57–59].

**Fig 2:**
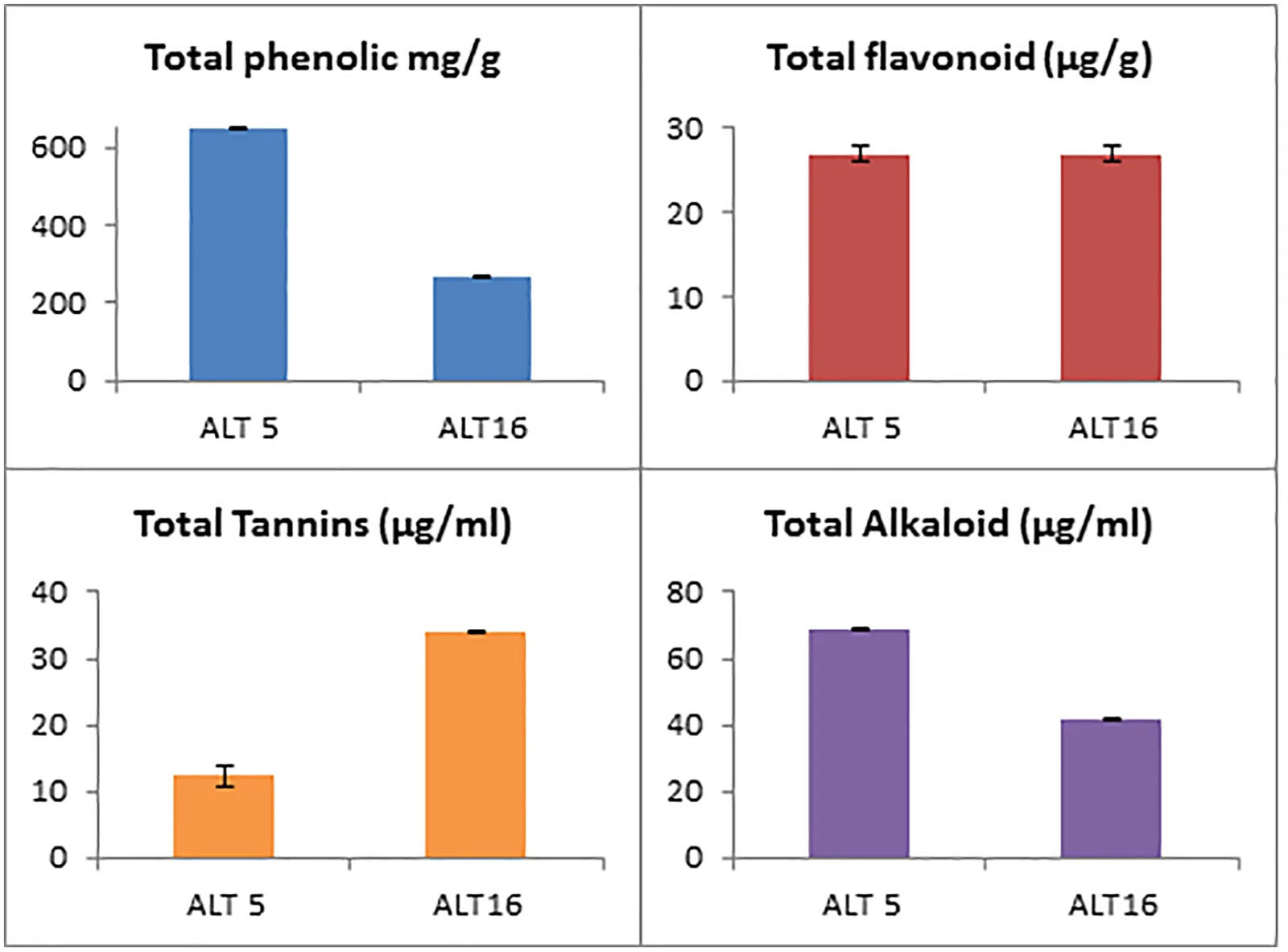
Phytochemical analysis of endophytic fungi. ALT 5:and ALT16 = *Alternaria* sp*.* and *A. alternata.*

### 3.3. GC-MS

Results in Table 1 and Fig 3 A&B revealed that endophytic fungi *Alternaria* sp*.* and *A. alternata* have different biological activities due to the creation of numerous secondary metabolites. The results showed that some extra-secondary metabolites identified in ethyl acetate *Alternaria sp extract,* contained 10 bioactive compounds. The major component being oleic acid (24.67%), palmitic acid (Hexadecanoic acid) (15.31%), stearic acid (13.65%), cis-Vaccenic acid (5.27%), trans-9-Octadecenoic acid, Pentyl ester (3%), androstan-17–one,3–ethyl-3–hydroxy-, (5à)-(2.38%) that represent highly antimicrobial, antifungal, anticancer, and anti-inflammatory activity. Moreover, *A. alternata* produced 9 bioactive compounds with major components: oleic acid (25.04%), stearic acid (11.3%), a-amyrin (10.95%), palmitic acid (Hexadecanoic acid) (6.19%), and Isochiapin B (4.77%). These results agree with previous studies that reported ethyl acetate of endophytic fungi containing numerous biologically active components, including oleic acid, Hexadecanoic acid, stearic acid, Octadecenoic acid, and Isochiapin-B [41]. Oleic acid is considered a vital anti-inflammatory agent alongside its anti-cancer activity, antiallergenic and pesticidal stuffs [60]. Hexadecanoic acid has great bactericidal influence against bacterial human pathogens [61]. Stearic acid has bactericidal effects against human pathogenic bacteria, including *Listeria monocytogenes* and *Staphylococcus aureus*, at low concentrations [62]. Phthalic acid esters have commonly been applied in frequent customer products, including therapeutic deliveries, cosmetics, food containers, construction tools, and home-produced furnishings [63]. Amyrin has antidepressant and anti–inflammatory activity [64]. Isochiapin-B has cytotoxic properties in contradiction to Hepatocarcinoma cell lines, bactericidal activity, antioxidant activity, and antiviral actions [65]. GC–MS mass spectra of the major compounds identified in the fungal extract was showed in figures S1 and S2 Figs supplementary.

**Table 1:** The distinguished compounds through GC-MS of crude ethyl extracts of the endophytic *Alternaria.*

Compound name	RT (min)	Peak Area %		Activity	Ref.
		*Alternaria* sp.	*A. alternata*		
Palmitic acid	30.52	15.31	6.19	Antioxidant, antibacterial, anti-inflammatory	[66]
Stearic acid	32.09	13.65	11.3	Antioxidant, antimicrobial and anticancer	[67,68]
Oleic acid	33.99	24.67	25.04	Fungicidal activity	[69]
cis-Vaccenic acid	34.01	5.27	–	Antimicrobial	[70]
Androstan-17–one, 3–ethyl-3–hydroxy-, (5à)-	34.51	2.38	1.79	Antimicrobial activity	[71]
Trans-9-Octadecenoic acid, pentyl ester	35.18	3	1.40	Bactericidal, antioxidant	[68]
Eicosanoic acid	37.19	1.4	–	Antioxidant, Antimicrobial and anticancer	[72]
a-Amyrin	37.54	–	10.95	Anti–inflammatory and antioxidant, combat acute pancreatitis.	[73]
Octadecanoic acid (Z)-, 2,3-dihydroxypropyl ester	38.05	2.6	0.96	Anti-microbial Anti-oxidant	[74]
Glycidyl oleate	43.28	0.81	–	Antimicrobial, Antioxidant and Anticancer	[75]
Stigmast-5-en-3-ol, oleate	47.18	1.1	0.38	Antimicrobial and antioxidant	[76]
Isochiapin B	47.68	–	4.77	Insecticide and antitumor agents.	

**Fig 3:**
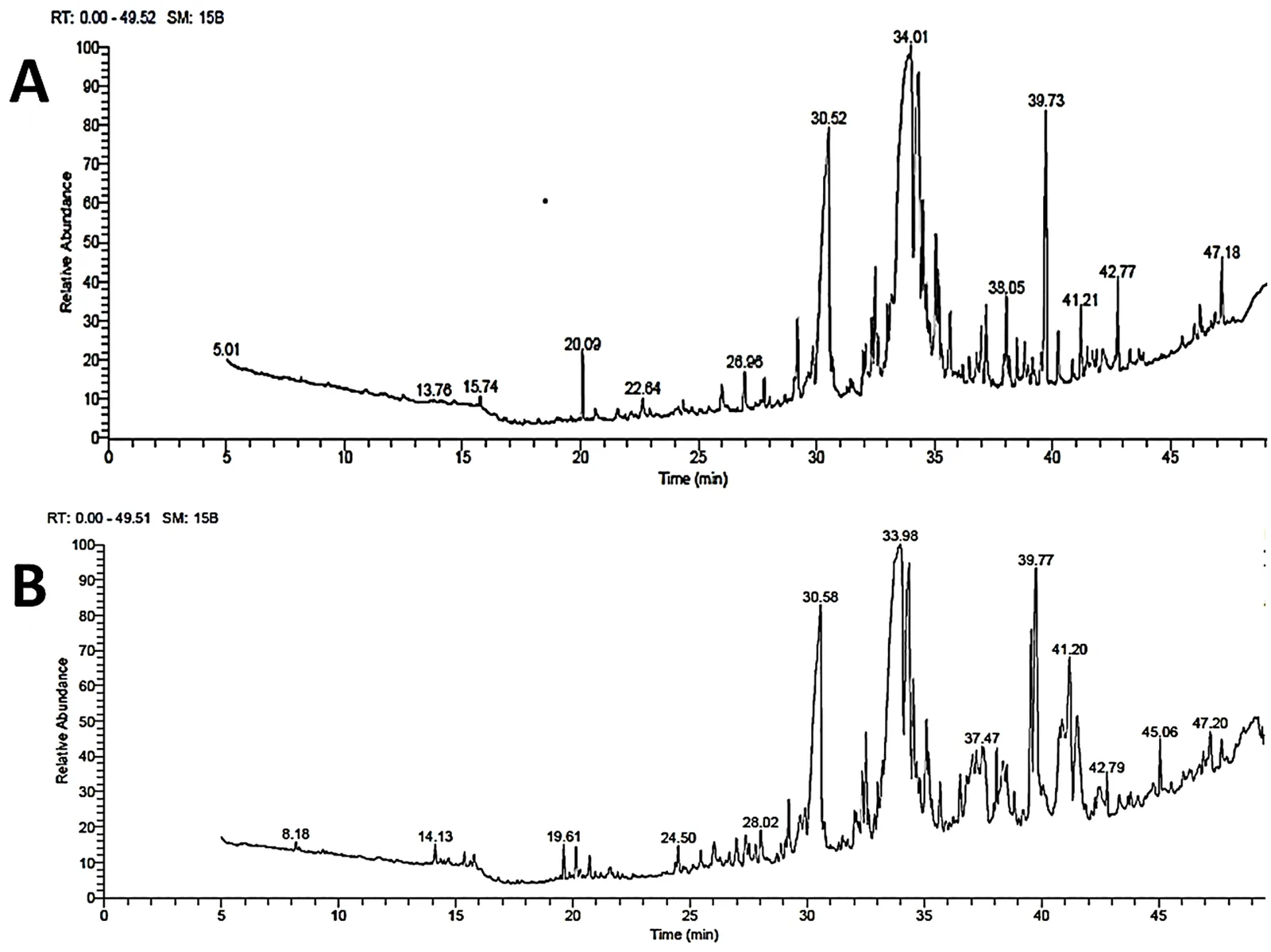
GC-MS of ethyl–acetate crude extracts of *Alternaria* sp. (A) and *A. alternata* (B).

### 3.4. Cytotoxicity and anticancer activity

The cytotoxicity of crude extracts of *Alternaria* sp*.* and *A. alternata* against vero cell line was evaluated as illustrated in Fig 4. Findings demonstrated that the cell viability of the crude extract of *Alternaria* sp*.* at 31.25, 62.5, 125, and 250 µg/ml was 99.78, 84.4, 69, and 51%, respectively. Moreover, the cell viability of the crude extract of *A. alternata* at 31.25, 62.5, 125, 250, 500, and 1000 µg/ml was 99.95, 99.86, 99.82, 99.78, 77, and 47%, respectively. Moreover, the IC_50_ for crude extracts of *Alternaria* sp*.* and *A. alternata* was 288 and 950µg/ml respectively. If the IC_50_ is equal to or more than 90μg/ml, the substance is considered non-cytotoxic [18,77]. On the other hand, crude extracts of *Alternaria* sp*. and A. alternata* displayed anticancer action toward the MCF 7 malignant cell-line, as illustrated in Fig 5. Extracts of *Alternaria sp.* and *A. alternata* exhibited anti–cancer efficacy against the MCF7 cell line, with the activity of *A. alternata* being higher than that of *Alternaria sp.,* according to the findings of the study. In addition, the findings demonstrated that the crude extract of *Alternaria* sp. exhibited anticancer activity at doses of 1000,500, 250,125, and 62.5 µg/ml, where anticancer activity was 86.3, 80.6, 71.0, 60.6, and 39.6%, respectively. As an additional point of interest, the crude extract of *A. alternata* demonstrated anticancer activity 98.0, 96.0, 89.3, 56.0, and 9.53% at doses 1000, 500, 250,125, and 62.5µg/ml respectively. The crude extracts of *Alternaria* sp*.* and *A. alternata* revealed that their IC_50_ values were 96.3 and 117.4 µg/ml, respectively, based on the data presented. An earlier study conducted by Wang, Ma [78] utilized endophytic *A. alternata* for the purpose of producing anticancer compounds. These compounds included dibenzo–α–pyrone derivatives and a benzo–γ–pyrone derivative.

**Fig 4:**
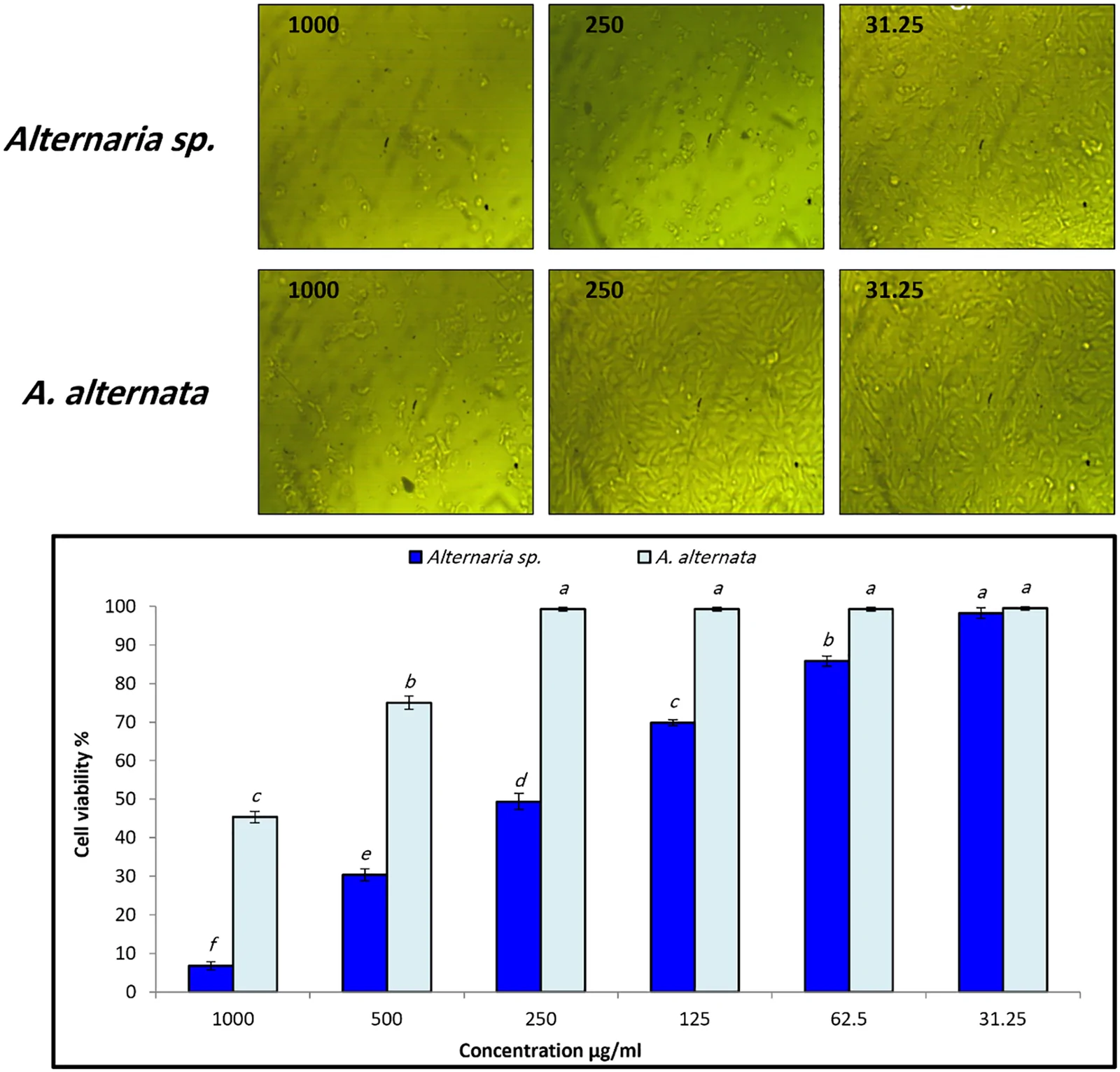
Cytotoxicity of crude extracts of *Alternaria* sp. and *A. alternata* toward Vero normal cell line. Letters *a, b, c,d, f, and e* = significance power.

**Fig 5:**
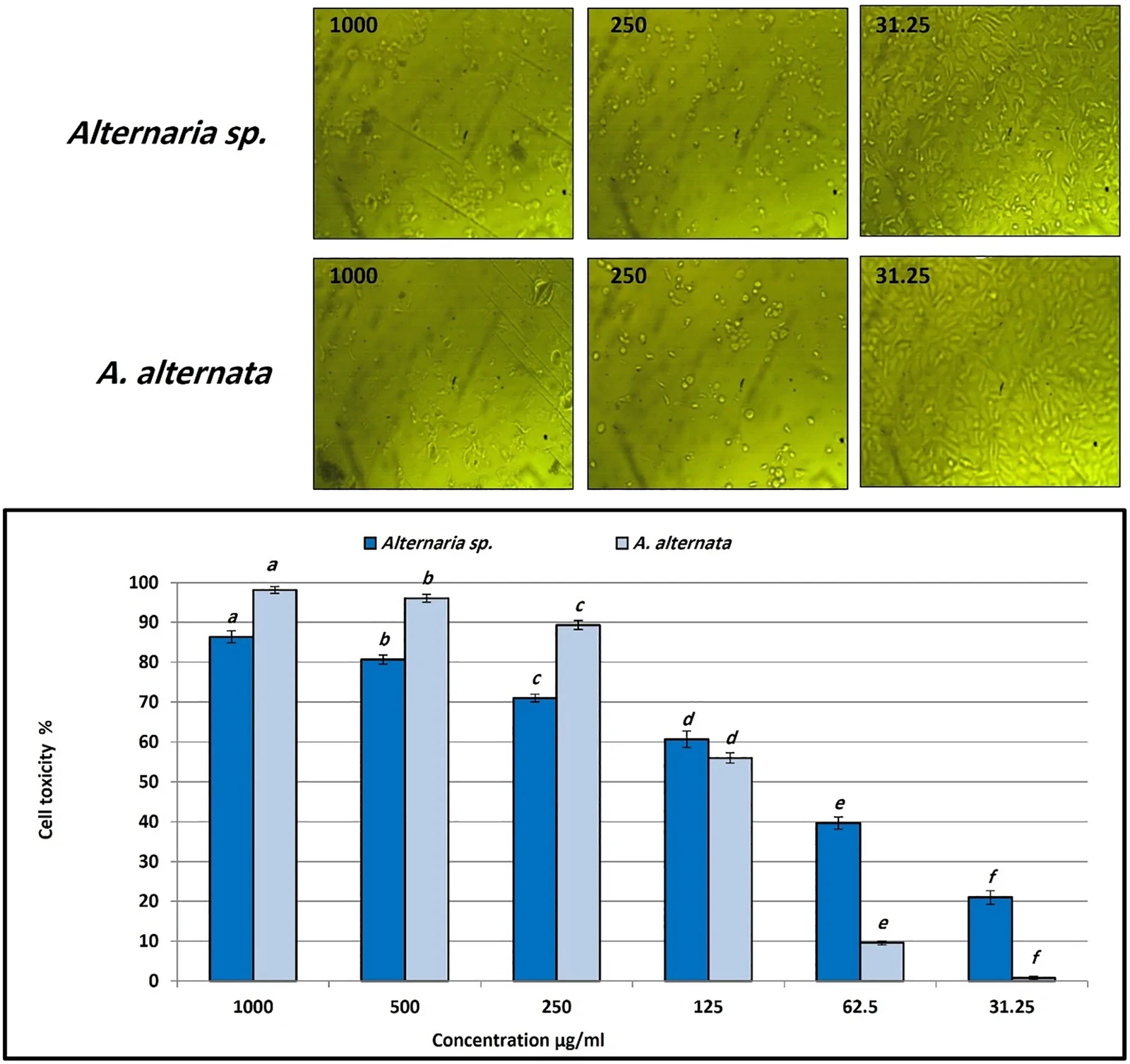
Anticancer activity of crude extracts of *Alternaria* sp. and *A. alternata* against MCF7 cancerous cell line. Letters a, b, c, d, e & f Mean significance power.

### 3.5. Antimicrobial activity

Fungal endophytes are recognized as a crucial source of bioactive substances with various biological effects, including antimicrobial and anti-cancer properties [79]. In the current study, crude extracts of *Alternaria* sp. and *A. alternata* were evaluated against pathogenic bacterial and fungal as shown in Table 2. The findings demonstrated that both crude extracts had potential anti–bacterial activity as well as anti–fungal efficacy. There was evidence that the crude extract of *Alternaria* sp. demonstrated antibacterial efficacy against *E. coli, P. aeruginosa, B. subtilis, and S. aureus.* The inhibition zones for these bacteria were 25.5±0.87, 15.7±1.53, 30.8±0.76, and 21.0±1.00 mm, respectively.

**Table 2:** Antimicrobial activity of crude extracts of *Alternaria* sp. and *A. alternata.*

Microbial strains	Diameter inhibition zones/ mm			
	DMSO	*Alternaria* sp.	*A. alternata*	AMC/Fluc
***E. coli***	ND	25.5 ± 0.87^b^	17.3 ± 1.43^c^	ND
***P. aeruginosa***	ND	15.7 ± 1.53^f^	11.9 ± 1.01^e^	ND
***B. subtilis***	ND	30.8 ± 0.76^a^	23.8 ± 1.31^a^	18.9 ± 1.20^a^
***S. aureus***	ND	21.0 ± 1.00^d^	15.5 ± 1.32^d^	9.5 ± 0.71^d^
***C. albicans***	ND	22.8 ± 1.04^c^	19.0 ± 0.95^b^	14.7 ± 0.92^b^
***C. neoformans***	ND	19.3 ± 0.58^e^	16.6 ± 1.10^cd^	13.0 ± 1.34^c^

ND: Not Detected. Letters a, b, c, and d = significance power

Similarly, the crude extract of *A. alternata* displayed antibacterial activity, but to a lower level *Alternaria* sp. *B. subtilis* had the maximum sensitivity, with an inhibition zone of 23.8±1.31 mm. *P. aeruginosa* had the lowest sensitivity, with an inhibitory zone of 11.9±1.01 mm. Standard AMC only showed antibacterial activity against Gram-positive bacteria, excluding others. It did not show efficacy against Gram-negative bacteria. This antibacterial activity, might be attributed to their capacity to deactivate microbial connections, damage bacterial membranes, and exhibit cell transference capabilities. They also contribute to the preservation of numerous enzymes linked with bacterial and viral pathogenicity. Furthermore, both extracts showed antifungal activity, with *Alternaria* sp. outperforming *A. alternata* in this regard. *Alternaria* sp’s crude extract showed good antifungal effectiveness against *C. albicans* and *C. neoformans*, with inhibition zones of 22.8±1.04 and 19.3±0.58 mm. The crude extract of *A. alternata* showed antifungal activity, with inhibition zones of 19.0 ± 0.95 and 16.6 ± 1.10 mm.

In a recent investigation, reported that an extract from endophytic *A. alternata* has antibacterial properties against *B. subtilis and S. aureus*. Furthermore, the *A. destruens* extract derived from Calotropis gigantean inhibits a variety of harmful microbial strains [35]*.* Caruso, Palombo [80] isolated *Alternaria* sp. from the *Eremophila longifolia* plant and tested its antibacterial activity. The extract was found to be effective against methicillin-sensitive *S. aureus* ATCC 25923, MRSA strains M173525, and M180920. Furthermore, a new molecule termed 2-Fluorobenzoic acid heptadecyl was isolated from endophytic *A. alternata* and demonstrated potential antibacterial action against multidrug resistant bacterial isolates [81]. Likewise, Endophytic *Alternaria* sp which isolated from *Salvadora persica* showed antibacterial activity toward *S. aureus and E. coli* Also, reported that, endophytic *Alternaria tenuissima PE2* exhibited inhibitory effect toward human-pathogenic *C. albicans*. Similarly, Endophytic *Alternaria* sp. isolated from *Salvadora persica* shown antibacterial activity against *S. aureus* and *E. coli* [82]. Furthermore, Chatterjee, Ghosh [83] found that endophytic *Alternaria tenuissima* PE2 inhibited human-pathogenic *C. albicans*.

### 3.6. Antioxidant activity

In the current study, the antioxidant activity of crude extracts of *Alternaria* sp. and *A. alternata* was evaluated at different concentrations (3.9–1000µg/ml) as shown in Fig 6. Results illustrated that, antioxidant activity of *Alternaria* sp. at doses of 1000,500, 250,125, 62.5,31.25, and 15.62 µg/ml was 82.3, 74, 51.3, 40.1, 10, and 1.6%, respectively. Furthermore, the antioxidant activity of *A. alternata* at doses of 1000,500, 250,125, 62.5,31.25, and 15.62 µg/ml was 89.6, 77.6, 65, 51.1, 39.4, 21.2, and 10.8%. Moreover, the EC50 of crude extracts of *Alternaria sp*. and *A. alternata* was 234.8 and 133.4 µg/ml, respectively. Phenolic compounds are responsible for this activity, as demonstrated by phytochemical assaying and GC–MS. Among endophytic *Alternaria* species, *A. alternata* is the most prevalent in producing antioxidant compounds. *Alternaria* sp. EA crude extract shown promising antioxidant activity in a prior investigation; the EC50 was 520 µg/ml [84]. An further investigation verified that the ethyl acetate extract of *Alternaria* sp. (ML4) shown a noteworthy reducing power activity and 85.20% DPPH scavenging activity at a dose of 300 µg/ml [85].

**Fig 6:**
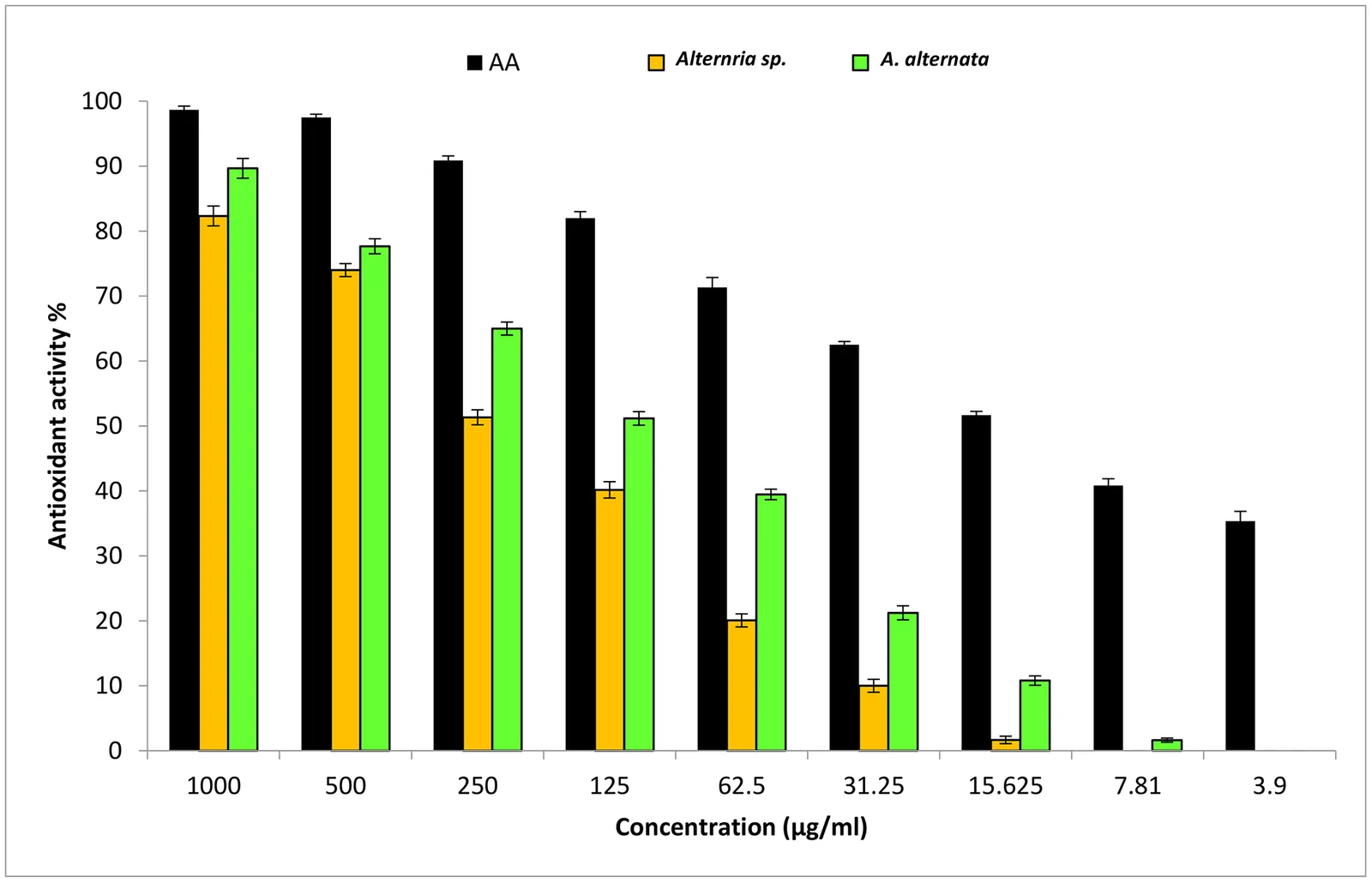
Antioxidant activity of fungal extracts of *Alternaria* sp. and *A. alternata* at different concentrations, AA = Ascorbic acid.

## 4. Conclusion

This is the first report of isolation and characterization of endophytic *Alternaria* species from *Reseda pruinosa* to our knowledge. Both *Alternaria* isolates were characterized to produce various secondary metabolites with high phenolics, flavonoids, tannins and alkaloids contents and linked to significant antioxidant, antimicrobial and selective anti-cancer activities. Our results highlighted the biotechnological and pharmaceutical potential of endophytic fungi associated with *R. pruinosa*, as a promising source of biologically active natural products. However, further studies are required to purify and identify the active components, to elucidate their molecular mechanisms of action, to evaluate their efficacy and safety by in vivo studies, and to optimize fermentation conditions for large-scale production.

## Supporting information

S1 Fig GC–MS mass spectra of the major compounds identified in the *Alternaria sp* extract. (PDF)

S2 Fig GC–MS mass spectra of the major compounds identified in the *Alternaria alternata* extract. (PDF)
