# Supplementary material for: Phytochemical analyses, antimicrobial, antioxidant and anticancer activities of endophytic fungi from leaves of Reseda pruinosa
Source: PLoS One. 2026 Oct 29;21(10):e0357606. doi: 10.1371/journal.pone.0357606 (PMC13718966; doi:10.1371/journal.pone.0357606)
Supplement: Supplementary file 2 [file pone.0357606.s002.pdf]

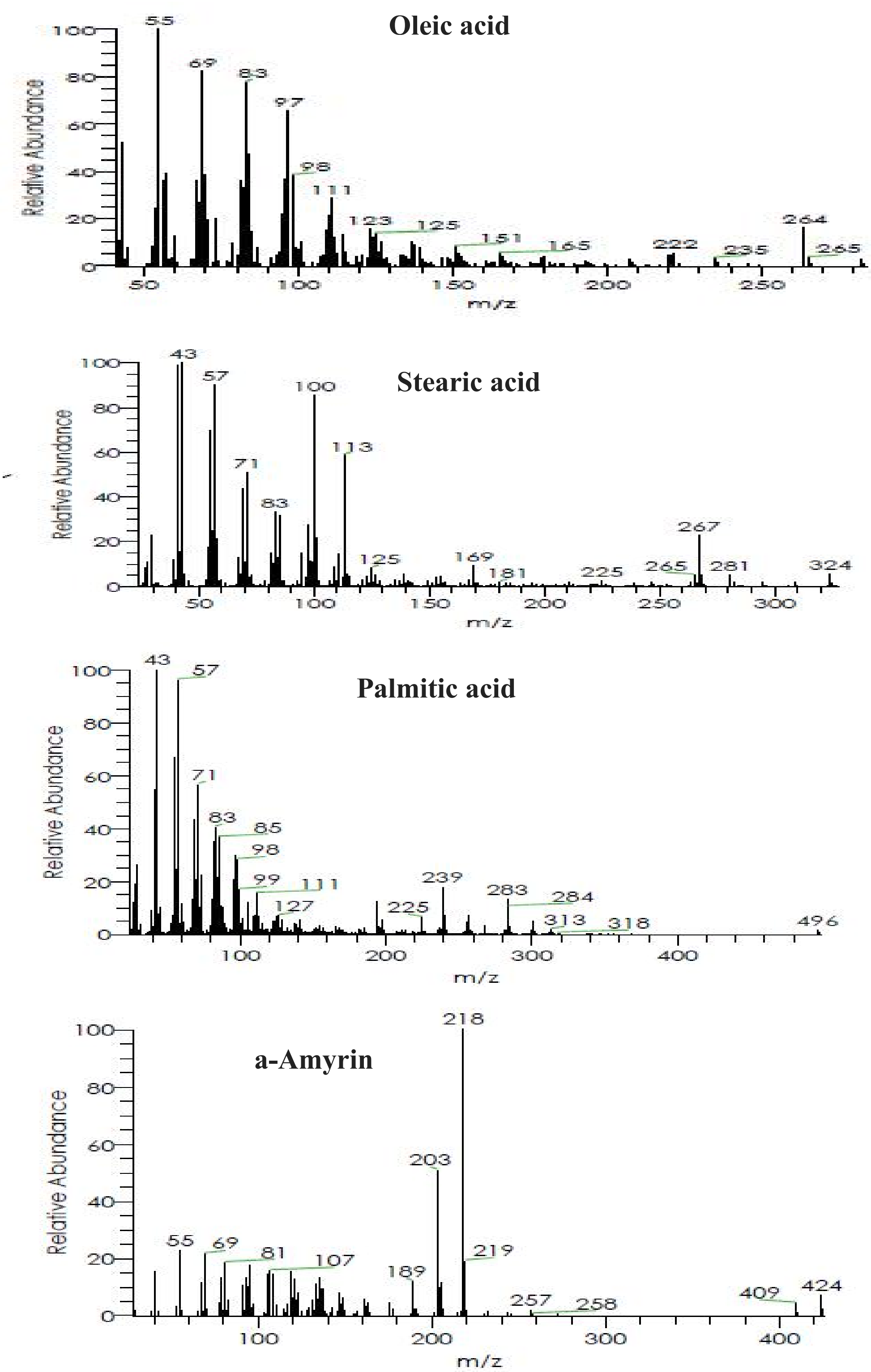

S2 Figure. GC–MS mass spectra of the major compounds identified in the *Alternaria alternata* extract.
